# Case Report: a rare pediatric case series of multiple endocrine neoplasia type 2B presenting with laryngotracheal involvement

**DOI:** 10.3389/fendo.2026.1837870

**Published:** 2026-07-24

**Authors:** Zhibo Xie, Jiarui Chen, Hongming Xu, Xiaoyan Li

**Affiliations:** Department of Otorhinolaryngology Head and Neck Surgery, Shanghai Children’s Hospital, School of Medicine, Shanghai Jiao Tong University, Shanghai, China

**Keywords:** laryngeal obstruction, medullary thyroid carcinoma, multiple endocrine neoplasia type 2B, neuroma, pediatrics

## Abstract

**Background:**

Multiple endocrine neoplasia type 2B (MEN2B) is a rare autosomal dominant genetic disorder caused by activating germline mutations in the *RET* proto-oncogene, typically arising during embryogenesis. The syndrome is characterized by the coexistence of medullary thyroid carcinoma, pheochromocytoma, gastrointestinal ganglioneuromas, and diffuse mucosal neuromas, the latter representing a pathognomonic clinical feature. In pediatric patients, mucosal neuromas most commonly involve the oral cavity, whereas laryngeal or other airway involvement is exceedingly rare and often underrecognized.

**Methods:**

From 2013 to 2025, two pediatric patients diagnosed with MEN2B were identified at our center. Both cases were confirmed to harbor pathogenic *RET* gene mutations by genetic testing.

**Result:**

In this study, we describe two pediatric cases of MEN2B in which laryngeal obstruction and progressive respiratory compromise were the initial clinical manifestations. Laryngoscope and CT examinations revealed mucosal neuromas within the laryngotracheal airway, and postoperative histopathology confirmed the diagnosis of neuroma. We further review the available literature on laryngotracheal involvement in MEN2B to elucidate its phenotypic spectrum and clinical implications. Timely recognition through detailed phenotypic assessment, molecular confirmation of *RET* mutations, and multidisciplinary management is essential to optimize outcomes.

**Conclusion:**

Laryngeal or airway neuromas are extremely rare. Clinicians should maintain a high index of suspicion for laryngeal neuromas in children presenting with characteristic craniofacial dysmorphism or a family history suggestive of endocrine neoplasia, as prompt genetic evaluation and integrated management may be life-saving.

## Introduction

Multiple endocrine neoplasia (MEN) comprises a group of disorders characterized by the development of tumors or hyperplastic lesions in two or more endocrine glands. These syndromes may occur sporadically or arise from inherited germline mutations, with MEN type 1 (MEN1) and MEN type 2 (MEN2) being the most extensively studied forms, each affecting approximately 1 in 30,000 individuals (1, 2). Although these conditions demonstrate high genetic penetrance, their clinical presentations are remarkably heterogeneous, often complicating early diagnosis. Advances in the identification of causative germline mutations—MEN1 mutations in MEN1 and *RET* mutations in MEN2—have substantially enhanced our understanding of the molecular mechanisms underlying endocrine tumorigenesis. These insights not only elucidate disease pathophysiology but also have practical clinical implications, enabling earlier diagnosis, improved risk stratification, and the implementation of individualized management strategies for both patients and at-risk family members (3).

MEN 2 is clinically classified into MEN 2A and MEN 2B. MEN 2A comprises four recognized phenotypes: classical MEN 2A, defined by the triad of medullary thyroid carcinoma (MTC), pheochromocytoma, and primary hyperparathyroidism; MEN 2A associated with cutaneous lichen amyloidosis; MEN 2A associated with Hirschsprung’s disease; and familial MTC, initially described as a thyroid-restricted form of diseases (4). MEN 2B is characterized by MTC and pheochromocytoma, accompanied by distinctive extra-endocrine manifestations, including ganglioneuromatosis of the aerodigestive tract, musculoskeletal abnormalities, and ocular defects (5, 6). The syndrome is most frequently driven by the *RET* M918T germline mutation, which accounts for approximately 95% of cases, while the A883F variant is responsible for fewer than 5% (7–9). Rare compound *RET* variants, such as E768D/L790F, V804M/Q781R, V804M/E805K, and V804M/Y806C, have also been reported in association with MEN 2B, highlighting the genetic heterogeneity underlying this aggressive syndrome.

Pediatric MEN 2B is primarily diagnosed through *RET* genetic testing, with the M918T mutation identified in approximately 95% of cases (10). Unlike adults, affected children often present earlier and with more pronounced clinical manifestations. MTC represents the defining feature and is frequently accompanied by pheochromocytoma. Characteristic extra-endocrine manifestations include ganglioneuromas of the aerodigestive tract, musculoskeletal abnormalities, ocular features, and a marfanoid habitus (11). At our center, we encountered two pediatric patients whose initial presentation was respiratory distress associated with varying degrees of laryngeal obstruction—a manifestation that is exceedingly rare, with only isolated cases reported worldwide. Here, we present a comprehensive characterization of these cases, together with a literature review, to further support the early recognition and diagnosis of pediatric MEN 2B.

## Case presentation

### Patient 1

**Figure 1:**
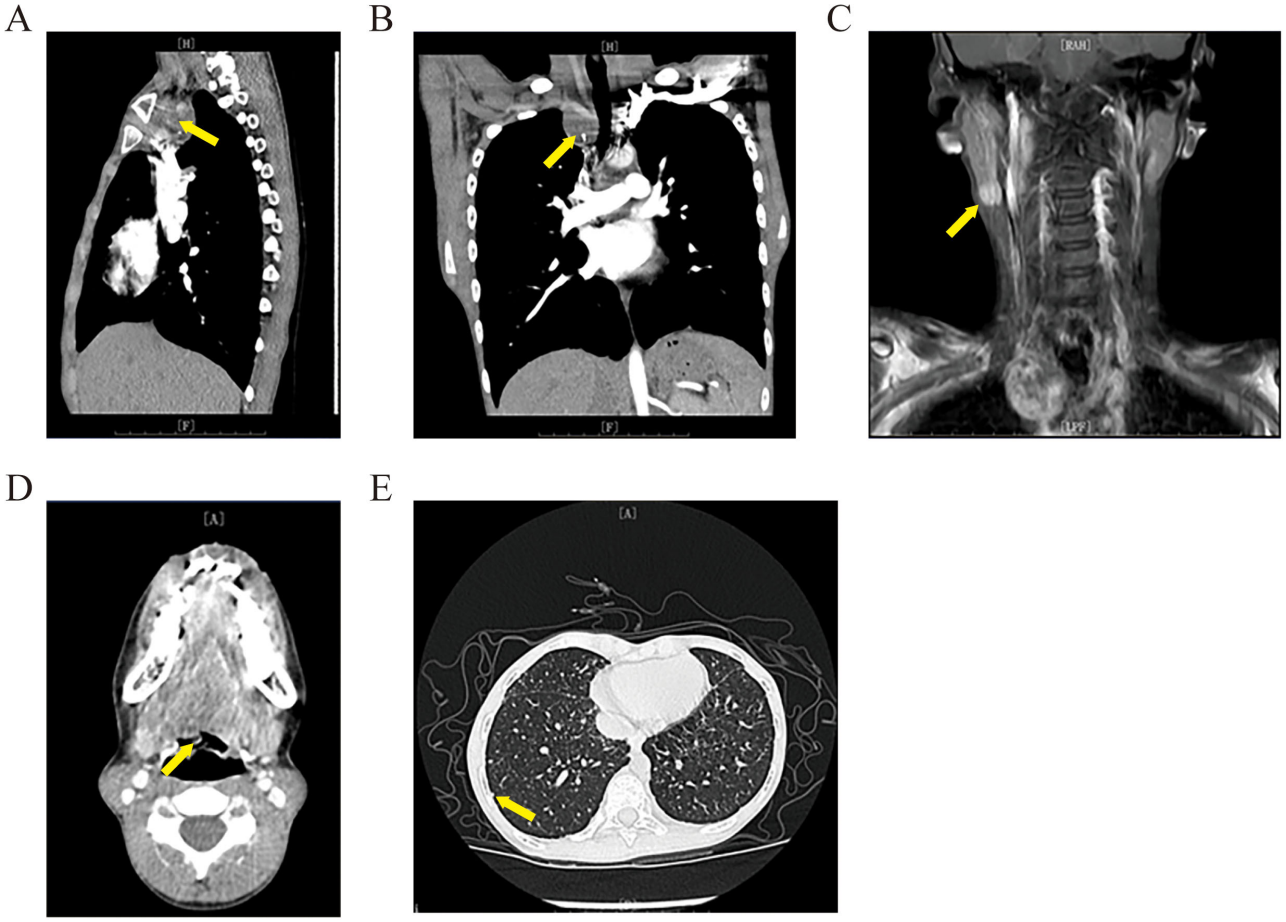
Clinical presentation of patient 1. **(A)** Sagittal CT image demonstrating mediastinal metastasis of medullary thyroid carcinoma in a pediatric patient following surgery; **(B)** Coronal CT image demonstrating mediastinal metastasis of medullary thyroid carcinoma in a pediatric patient following post-surgery; **(C)** Coronal MRI image demonstrating mediastinal and cervical lymph node metastasis of medullary thyroid carcinoma in a pediatric patient following surgery. **(D)** CT image showing a pharyngeal mass in a pediatric patient following surgery for medullary thyroid carcinoma. **(E)** CT image showing multiple pulmonary metastasis of medullary thyroid carcinoma in a pediatric patient following surgery.

The first case involved a 9-year-old girl who presented with labored breathing accompanied by hoarseness in 2017. At a local hospital, physical examination revealed a neck mass, which was suspected to be MTC (laryngoscopic examinations had been performed at that time, with no obvious subglottic lesions identified). Following surgical resection, the patient could not be extubated. Laryngoscopic examination demonstrated bilateral vocal cord paralysis, and a tracheostomy was subsequently performed. The patient later experienced recurrent tracheostomy tube obstruction, prompting referral to our center in 2018. Repeat laryngoscopy revealed persistent bilateral vocal cord paralysis and multiple subglottic masses. Physical examination identified bilateral cervical lymphadenopathy, and positron emission tomography computed tomography imaging suggested possible lymph node metastasis. The patient subsequently underwent bilateral cervical lymph node dissection and complete excision of the laryngeal mass at our institution Airway patency was the primary concern in this patient. A tracheostomy had been established preoperatively; therefore, the patient remained in a stable respiratory condition without evidence of hypoxemia. Preoperative assessment confirmed the absence of significant hypoxemia. During surgery, general anesthesia was maintained via an endotracheal tube inserted through the tracheostomy stoma, ensuring a secure airway and uninterrupted ventilation. The cuffed tube also prevented blood and secretions from entering the distal airway. At the end of the procedure, after thorough suctioning of tracheal secretions, the endotracheal tube was removed and replaced with a tracheostomy cannula to restore the preoperative airway configuration. The patient was then transferred to the intensive care unit for close postoperative monitoring, and no postoperative airway obstruction was observed Histopathological examination confirmed metastatic MTC in the cervical lymph nodes, while the laryngeal lesion was identified as a neuroma. Gene testing revealed a *RET* M918T mutation, confirming a pathogenic variant consistent with MEN 2B. Further inquiry revealed no known family history of MEN2B or other endocrine neoplasms. Postoperatively, the patient intermittently tolerated tracheostomy tube occlusion. However, one year later, she developed recurrent dyspnea and dysphagia. Follow-up CT imaging demonstrated multiple bilateral cervical and mediastinal lesions consistent with tumor recurrence and metastasis (**Figure 1**). Despite repeated surgical resection of the cervical and mediastinal masses, the patient succumbed to disease progression three months after the final operation in 2024 (The patient’s overall survival after diagnosis was approximately 7 years).

### Patient 2

**Figure 2:**
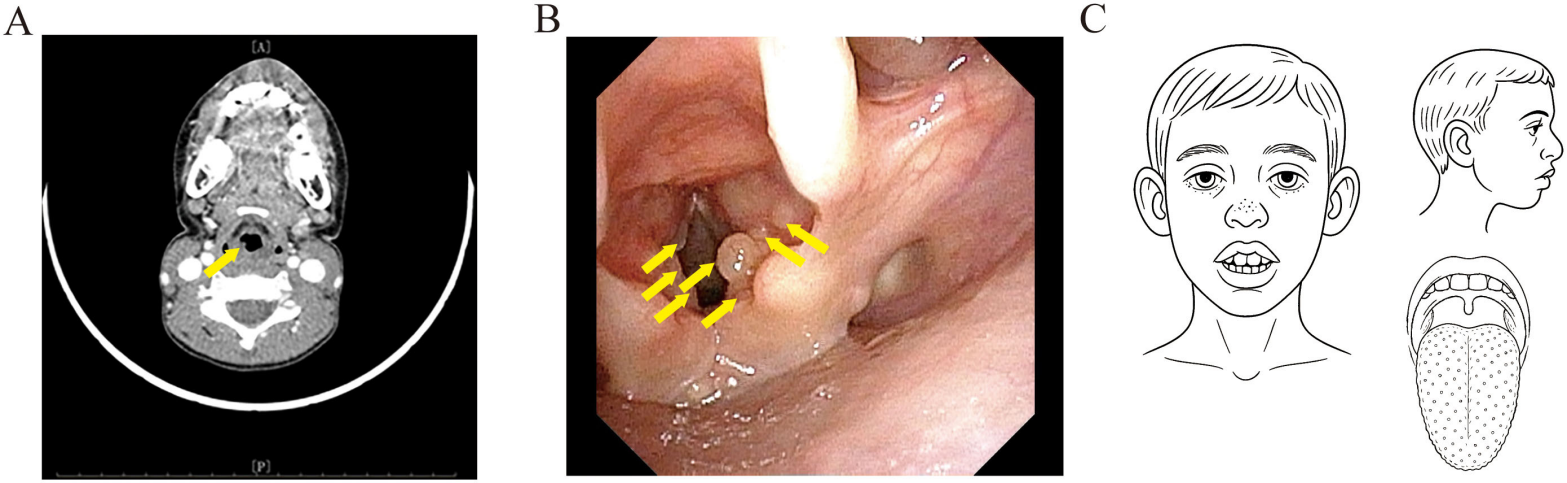
Clinical presentation of patient 2. **(A)** CT imaging showing multiple pharyngeal masses in a pediatric patient; **(B)** Laryngoscopic image demonstrating multiple pharyngeal masses in a pediatric patient; **(C)** Characteristic phenotypic features of MEN2B, including an elongated facial appearance, thickened eyelids, thickened and “blubbery lips”, prognathism, and a relatively flat nasal bridge. Oral findings include a broad tongue with multiple mucosal neuromas on the tongue, lips, and buccal mucosa, resulting in a nodular and irregular surface.

The second patient was a 6-year-old girl who initially presented with respiratory discomfort accompanied by mild pharyngeal irritation during swallowing in 2025. The patient also exhibited mild hoarseness. During quiet respiration, faint coarse breath sounds were audible without apparent inspiratory retraction. However, with exertion, the patient developed mild respiratory distress characterized by increased respiratory effort and occasional deep, labored breathing. Flexible laryngoscopy performed at our outpatient clinic revealed a supraglottic mass, which was further confirmed by CT to involve both the supraglottic and glottic regions (**Figure 2**). Thyroid ultrasonography revealed multiple nodules with concomitant cervical lymphadenopathy. Preoperative polysomnography showed features of upper airway obstruction, including intermittent hypoxemia and episodes of apnea, with an apnea–hypopnea index (AHI) of 1.5 and a nadir oxygen saturation of 85%. Intraoperatively, high-frequency jet ventilation system was used to maintain adequate oxygenation and airway patency. Lesion resection was performed using a plasma radiofrequency device, allowing precise excision of the laryngotracheal mass while avoiding interference from a conventional endotracheal tube within the glottic inlet. Postoperatively, both dyspnea and dysphagia improved significantly. Histopathological examination confirmed a neuroma. Genetic testing identified a *RET* M918T mutation. Based on the combined clinical, radiological, genetic, and pathological findings, the patient was diagnosed with MEN2B. A detailed family history revealed no known history of MEN2B or related endocrine tumors. At 3 months postoperatively, follow-up neck ultrasonography demonstrated enlargement of bilateral thyroid nodules and cervical lymphadenopathy, accompanied by elevated serum calcitonin levels. Fine-needle aspiration at a local hospital suggested the possibility of MTC. The patient subsequently underwent total thyroidectomy with bilateral cervical lymph node dissection, which confirmed progression to MEN2B-associated MTC. Postoperatively, the patient underwent regular follow-up, including neck ultrasonography, thyroid function tests, and measurements of serum tumor markers, including calcitonin and carcinoembryonic antigen (CEA). As of June 2026, no evidence of MTC recurrence has been observed.

## Discussion

MEN2B is a rare autosomal dominant disorder caused by activating mutations in the *RET* proto-oncogene, most commonly involving codon M918T. This syndrome is characterized by the early development of MTC, pheochromocytomas, and multiple mucosal neuromas. While the oral and lingual mucosa are the most frequently affected areas, involvement of the larynx and airway has been increasingly recognized, particularly in pediatric patients. Airway involvement may pose significant diagnostic and management challenges (4).

In MEN2B, airway involvement typically manifests as multiple mucosal neuromas affecting the laryngeal, glottic, or subglottic regions (12). These lesions result from hyperplasia of Schwann cells and ganglion cells within the submucosa, leading to nodular or polypoid mucosal thickening (13). Clinically, they are often asymptomatic in the early stages and may be incidentally detected during routine examination. However, as lesions enlarge or depending on their location, they may cause progressive dysphonia, hoarseness, dyspnea, or even airway obstruction. In pediatric patients, these symptoms are frequently misinterpreted as common airway diseases such as laryngomalacia, croup, or asthma, which may lead to delayed diagnosis.

Endoscopic evaluation and radiologic imaging play essential roles in the diagnosis of airway involvement in MEN2B. Laryngoscopy enables direct visualization of mucosal neuromas, which typically appear as smooth, sessile, or nodular lesions along the vocal cords, aryepiglottic folds, or subglottic region (14). High-resolution CT or MRI provides additional information regarding the extent of airway involvement, and helps exclude deep infiltration into adjacent structures. Histopathological examination combined with immunohistochemical staining remains the gold standard for diagnosis, with characteristic findings including disorganized nerve fiber bundles and ganglion cells, as well as positive staining for S-100 protein and neuron-specific enolase.

We carefully reviewed the studies *via* Pubmed, and identified 10 case reports worldwide (12, 14–22). Although mucosal neuromas frequently involve the lips, tongue, and buccal mucosa in MEN2B, laryngeal involvement has been rarely reported. Patient age varied from 3 months to 58 years. The *RET* M918T mutation was the most common reported variant. Among 10 cases, neuromas were most frequently localized to the vocal cords (7 cases) (**Table 1**). The predominant presenting symptoms are dysphonia, and hoarseness. Long-term follow-up demonstrated complete symptom resolution after surgical resection in most cases, although one pediatric patient developed a recurrent neuroma that remained clinically stable without progression. Compared with previously reported cases, our series provides several important clinical insights. To our knowledge, this is one of the few pediatric MEN2B case series in which laryngotracheal symptoms were the initial presenting manifestations, leading patients to seek otolaryngologic evaluation before the diagnosis of MEN2B was established. This finding suggests that airway involvement may precede recognition of characteristic endocrine manifestations of the syndrome.

**Table 1:** Studies on airway involvement in MEN2B patients.

Study ID	Age at onset (yrs)	Gender	Genetic mutation	Craniofacial features	Clinical features
Kudo et al.	20	F	RET	N/A	A left anterior neck mass led to the identification of bilateral arytenoid mucosal neuromas
Ghosh et al.	11	F	RET M918T	Coarse facies: a small nose, long philtrum, and prominent lips	Incidental laryngeal mucosal lesions and M918T-positive vocal cord nodules recurred after surgical excision but subsequently remained stable during follow-up
Qualia et al.	3-month	M	RET	N/A	Gastrointestinal pseudo-obstruction in infancy followed by MEN2B diagnosis at age seven due to oral mucosal neuromas; subsequent post-thyroidectomy dysphonia revealed a left vocal cord neuroma
Soroa-Ruiz et al.	28	F	RET	N/A	Developed intermittent dysphonia due to bilateral vocal cord neuromas, which did not recur after surgical excision.
Tolkemitt et al.	58	M	RET	N/A	A patient with progressive hoarseness after total thyroidectomy was found to have nodular lesions involving the right arytenoid cusps and aryepiglottic folds, with near-complete recovery of voice function four weeks after surgical excision
Lesourd et al.	28	F	RET	N/A	Multiple mucosal neuromas involving the oral cavity, nasopharynx, and larynx were identified at post-mortem examination in a patient who died of metastatic medullary thyroid carcinoma
McClurg et al.	30	F	RET	N/A	Dysphagia, dysphonia, and persistent cough were associated with multiple mucosal nodules involving the glottic and supraglottic regions of the larynx
Chornenkyy et al.	6	M	RET M918T	N/A	A patient carrying a prenatal RET M918T mutation underwent prophylactic thyroidectomy revealing medullary microcarcinoma and later developed eosinophilic esophagitis and bowel ganglioneuromas requiring colectomy. Surveillance endoscopy identified a partially obstructive left vocal cord ganglioneuroma, and subtotal excision resulted in minimal regrowth without symptoms on follow-up
Daou et al.	5	F	RET M918T	N/A	A patient presented with medullary thyroid carcinoma and extensive lymphadenopathy requiring tumor debulking and neck dissection. Postoperatively, severe stridor led to the identification and excision of bilateral S-100-positive mucosal neuromas on bronchoscopy
Alsalem et al.	Adolescent	M	RET M918T	N/A	A patient developed dysphonia, nocturnal stridor, and exertional dyspnea after thyroidectomy for medullary thyroid carcinoma with recurrent laryngeal nerve sacrifice, a left vocal process lesion was debulked and confirmed as a mucosal neuroma

Regarding treatment, the management approach is primarily determined by the severity of airway involvement. Asymptomatic or minimally symptomatic neuromas may be managed with close monitoring, whereas lesions causing significant airway obstruction often require microlaryngoscopic excision or partial resection to restore airway patency (18). In patients with MEN2B presenting with laryngotracheal involvement and clinically significant respiratory compromise, surgical decision-making is critical. When dyspnea occurs, the underlying etiology should be carefully evaluated. If respiratory distress is primarily caused by a large tumor producing extrinsic airway compression, the lesion should be thoroughly assessed. When complete excision is technically feasible and considered safe, prompt radical resection should be performed. If complete resection is not feasible, debulking surgery may be considered, with concurrent assessment of respiratory status to determine the need for tracheostomy. Further management should be guided by the histopathological characteristics of the lesion If respiratory compromise is not solely attributable to tumor compression, but instead results from tumor invasion or recurrent laryngeal nerve injury following surgery for MTC, leading to vocal cord paralysis, emergent endotracheal intubation should be performed to secure the airway and maintain vital functions. In cases where extubation is not achievable, tracheostomy should be considered to ensure long-term airway patency. In addition to standard otolaryngological management, a multidisciplinary approach-including pulmonology consultation and airway stenting, may provide symptomatic relief in selected cases. It should be emphasized that in airway-compromising tumors, particularly malignant lesions such as MTC, debulking procedures must be undertaken with caution. The indications, necessity, and safety of surgical intervention should be carefully evaluated on a case-by-case basis. In our case series, Patient 1 developed bilateral vocal cord paralysis following surgery for MTC, resulting in failed extubation and necessitating tracheostomy. Postoperatively, the patient intermittently tolerated tracheostomy tube occlusion. However, due to recurrent and metastatic progression of MTC, the patient subsequently developed airway compression and recurrent respiratory distress. CT revealed mediastinal metastasis and multiple cervical lymph node metastasis. Accordingly, a multidisciplinary surgical approach involving cardiothoracic surgery and other relevant specialties was undertaken to relieve airway obstruction. It is noteworthy that airway management in patients with MEN2B involving the respiratory tract may be particularly challenging during anesthesia. In some cases, large neuroma-like lesions may impede tracheal intubation. Therefore, preparation of HFJV equipment and other advanced airway management strategies is essential to ensure safe airway control.

Previous functional studies have proposed a cis-acting compound mutation model in which two kinase-domain *RET* variants located on the same allele synergistically enhance transforming activity (23–25). Within this framework, the E768D/L790F variant combination, located in the tyrosine kinase domain, may partially relieve autoinhibitory constraints, resulting in intermediate activation of downstream MAPK and PI3K/AKT signaling pathways. In contrast, the V804M/Y806C combination appears to exert a more pronounced structural impact, potentially altering kinase domain conformation and shifting critical regulatory dependencies, thereby leading to ligand-independent hyperactivation of *RET* signaling. Functionally, these distinct configurations are consistent with a spectrum of *RET* activation states, ranging from intermediate to MEN2B-like signaling intensity. This likely reflects conformational remodeling of the kinase domain, which alters key tyrosine residue dependence and enhances downstream signal transduction. Importantly, unlike classical two-hit tumor suppressor models, both variants reside on the same allele and act cooperatively in a synergistic, non-additive manner to increase receptor activation.

According to the *Chinese Experts’ Consensus on diagnosis and Treatment of medullary thyroid Carcinoma* (26), early *RET* genetic testing and family screening are strongly recommended in children with suspected or confirmed MEN2B to enable timely diagnosis and risk stratification. The consensus recommends a “prophylactic surgery-first” strategy for pediatric patients carrying high-risk *RET* mutations such as M918T. Given the high risk of early metastasis in MEN2B-associated MTC, prophylactic total thyroidectomy is recommended early in life (typically in infancy or early childhood, with earlier intervention considered in selected high-risk cases) to minimize disease development and progression. Preoperative evaluation should routinely include serum calcitonin and CEA levels to assess for occult MTC. Markedly elevated calcitonin levels or imaging evidence of thyroid nodules or local invasion may indicate clinical MTC, in which case more extensive surgical management, including central neck dissection, may be required to improve the likelihood of cure. In addition, the consensus emphasizes that children with MEN2B are at risk of concomitant pheochromocytoma; therefore, adrenal functional assessment and imaging evaluation must be completed prior to thyroid surgery to avoid perioperative hypertensive crises caused by undiagnosed catecholamine-secreting tumors. Given the systemic nature of MEN2B, a multidisciplinary approach, involving endocrinology, otolaryngology, and genetics specialists, is essential. Moreover, meticulous perioperative airway management is critical to optimizing surgical outcomes.

## Conclusion

In conclusion, although airway involvement in pediatric MEN2B is rare, it represents a clinically significant concern with potentially life-threatening consequences. Clinicians should maintain a high index of suspicion for laryngeal neuromas in children presenting characteristic craniofacial dysmorphism or a family history suggestive of endocrine neoplasia, as prompt genetic evaluation and integrated management may be life-saving. Raising awareness among pediatricians and otolaryngologists is essential to improve the early recognition and outcomes of this rare but clinically important manifestation of MEN2B.

## Data Availability

The raw data supporting the conclusions of this article will be made available by the authors, without undue reservation.
